# Effect of traumatic brain injury on the trough concentration of linezolid in patients with hospital-acquired pneumonia

**DOI:** 10.1038/s41598-025-28571-9

**Published:** 2025-12-29

**Authors:** Ying Zhang, Lian Tang, Mian Ma, Zhiwei Zhuang, Jinhui Xu, Lu Shi, Qian Zhang, Yanxia Yu, Zicheng Yu, Guangjuan Xu

**Affiliations:** 1 Department of Pharmacy, Suzhou Research Center of Medical School,Suzhou Hospital, Affiliated Hospital of Medical School, Nanjing University, Suzhou, China; 2 School of Pharmacy, Nanjing Medical University, Nanjing, China; 3 Gusu School, Nanjing Medical University, Suzhou, China; 4 Department of Pharmacy, The Affiliated Suzhou Hospital of Nanjing Medical University, Suzhou Municipal Hospital, Suzhou, China; 5 Department of neurosurgery, The Affiliated Suzhou Hospital of Nanjing Medical University, Suzhou Municipal Hospital, Suzhou, China; 6 Emergency Intensive Care Unit, The Affiliated Suzhou Hospital of Nanjing Medical University, Suzhou Municipal Hospital, Suzhou, China; 7 Institute of Clinical Pharmacology, Yangpu Hospital, Tongji University School of Medicine, Shanghai, China

**Keywords:** Traumatic brain injury, Linezolid trough concentration, Gram-positive bacteria eradication, Linezolid-induced thrombocytopenia, Antimicrobials, Antibiotics

## Abstract

To investigate the effect of traumatic brain injury (TBI) on linezolid trough concentration (C_min_) and Gram-positive bacteria eradication in patients with hospital-acquired pneumonia (HAP). HAP patients who treated with linezolid were collected and divided into TBI and non-TBI groups. Differences in the linezolid C_min_, Gram-positive bacterial eradication, clinical treatment success, and adverse reactions were analyzed between the two groups. A total of 177 patients were enrolled in the study, including 57 with TBI and 120 without TBI. After 1:1 propensity score matching (PSM), 46 patients with well-balanced baseline levels were enrolled. Linezolid C_min_ and Gram-positive bacterial eradication rate were lower in TBI group than in non-TBI group [2.06 (0.89, 6.89) mg/L, 6.70 (3.09, 13.48) mg/L, *P* < 0.001; 69.0% vs. 90.9%, *P* = 0.029]. Linezolid-induced thrombocytopenia (LIT) and severe LIT occurred in fewer patients in TBI group than in non-TBI group (4.3% vs. 28.3%, *P* = 0.005; 2.2% vs. 17.4%, *P* = 0.035). The linezolid C_min_ for the predicted eradication of Gram-positive bacterial was 1.62 mg/L. Further studies have found that the use of mannitol, glycerin fructose and high fluid intake and output may be responsible for the low C_min_ of linezolid in TBI group. Patients in TBI group had lower linezolid C_min_ and Gram-positive bacterial eradication rates compared with those in non-TBI group. A lower C_min_ level may predict a decrease in Gram-positive bacterial eradication rates, and patients with concomitant TBI may need to explore higher linezolid doses.

**Supplementary Information:**

The online version contains supplementary material available at 10.1038/s41598-025-28571-9.

## Introduction

Traumatic brain injury (TBI) is damage to the brain caused by external trauma. Patients with TBI often suffer from post-traumatic disturbances of consciousness and require endotracheal intubation and mechanical ventilation, which increases the risk of hospital-acquired pneumonia (HAP)^1^. Staphylococcus aureus, in particular methicillin-resistant Staphylococcus aureus (MRSA), is the most common pathogen causing pneumonia in TBI patients, accounting for 29–35%^2,3^. Linezolid has excellent antibacterial activity against MRSA and has shown superior penetration into the lung epithelial lining fluid and lung macrophages^4,5^. It is recommended by the Infectious Diseases Society of America (IDSA) as a first-line treatment for MRSA pneumonia^6^.

The recommended dose of linezolid in the instructions is 600 mg q12h, and the amount of exposure in vivo exposure is strongly correlated with its clinical efficacy and adverse reactions. Currently, the recommended steady-state trough concentrations (C_min_) of linezolid is in the range of 2–7 mg/L^7,8^. Previous studies have shown that factors such as renal impairment or dysfunction, hepatic impairment, and low or high body weight significantly contribute to its pharmacokinetic (PK) variability^9–12^. This variability may result in either increased or decreased exposure to linezolid, potentially leading to adverse effects such as thrombocytopenia or clinical treatment failure^7^. We have found that TBI patients have lower plasma concentrations of linezolid than other patients, which may increase the risk of treatment failure. TBI-induced cellular and biochemical cascades can lead to secondary tissue damage, such as disruption of renal autoregulation and alterations in hepatic drug metabolism^13^. In addition, in our patient population, there exist such factors as hyperdynamic states, cerebrospinal fluid (CSF) drainage or the influence of concurrent medications (e.g. vasopressors, diuretics)^14,15^. These pathophysiological changes may increase the volume of distribution and clearance of antimicrobial agents, resulting in lower exposure, as reported in previous studies treated with vancomycin^16^ and meropenem^17^. Luque et al.^15^ found a significant increase in the volume of distribution and clearance rate of linezolid in neurosurgical patients, which was differently with other populations.

The aim of this study was to investigate the effect of TBI on the plasma trough concentrations and microbiological efficacy of linezolid at the normal dose in patients with HAP, and to explore the possible influencing factors leading to the pharmacokinetic variability of linezolid in the patients with TBI.

## Methods

### Study design and population

This two-center study was conducted in the intensive care unit (ICU) and emergency intensive care unit (EICU) of Affiliated Suzhou Hospital of Nanjing Medical University and Suzhou Science and Technology Town Hospital. Critically ill patients treated with linezolid between July 2020 and June 2022 were retrospectively collected. The inclusion criteria were as follows: (1) age ≥ 18 years; (2) the clinical diagnosis was hospital acquired pneumonia^6^; (3) receiving linezolid therapy for at least 3 days, and the first course of linezolid treatment was included if there was more than one course of linezolid therapy; (4) the C_min_ of linezolid was determined; (5) patients with TBI admitted to the EICU with a Glasgow Score of 5 to 8 and diagnosis was confirmed by imaging tests such as cranial computed tomography (CT) and magnetic resonance imaging (MRI). Exclusion criteria were as follows: (1) baseline platelet count < 75 × 10^9^/L; (2) hemodialysis, continuous renal replacement therapy (CRRT), or extracorporeal membrane oxygenation during linezolid treatment; (3) concomitant disseminated intravascular coagulation or other bleeding disorders; (4) concomitant use of heparin or anti-platelet agents; (5) severe hepatic impairment; (6) linezolid C_min_ outside the limits of detection or incorrect sampling timing; (7) incomplete or missing demographic and clinical information.

### Data collection

Demographic and clinical data were collected for each patient by searching the hospital information system and the following information was recorded: (1) demographic characteristics include sex, age, weight, underlying diseases, and hospitalization time; (2) diagnosis, comorbidities, complications and fluid balance; (3) the type of infection, microbiological isolates and minimum inhibitory concentration (MIC); (4) medication regimen including dose, intervals, duration of linezolid administration, Steady-state trough concentration, concomitant antibiotic therapy and the use of dehydrating agents (e.g., mannitol, glycerin fructose); (5) laboratory indexes: the complete blood count (CBC) includes white blood cell (WBC) counts, neutrophil percentage (N%), Platelet (PLT) counts; serum inflammatory biomarkers include C-reactive protein (CRP), procalcitonin (PCT); biochemical indexes include hemoglobin (HB), total bilirubin (TBIL), albumin (ALB), alanine aminotransferase (ALT), creatinine (Cr); estimated glomerular filtration rate (eGFR) was measured within 1–3 days of linezolid administration and within one week of discontinuation of the drug. Laboratory indexes were tested every 3–5 days; (6) linezolid-related adverse reactions and treatment outcomes (28-day survival rate). An EXCEL database was set up for two-person entry and verification. The eGFR was calculated using the 2021 CKD-EPI equation^18,19^.

### Grouping and dosage regimen

Patients enrolled were classified into TBI and non-TBI groups. TBI is a type of brain injury caused by an external force. TBIs can include penetrating injuries (in which an object breaches the skull and dura, with direct damage to the brain parenchyma) and closed-head injuries (in which the skull and dura remain intact). All patients with TBI were confirmed by CT or MRI to have skull fractures, brain bruising, bleeding, or swelling. The initial dose of linezolid in all patients was 600 mg q12h, after which the dose was adjusted based on C_min_ and efficacy assessment. Only the first determination of linezolid C_min_ was included in our study for statistical analysis.

### Determination of linezolid C_min_

Blood samples (1 mL) were obtained and placed in yellow vacuum sampling vessel (containing coagulant and separation glue) within 30 min before the fifth dose^20^, centrifuged within 2 h, and then 0.5 mL supernatant was collected and placed in the refrigerator at − 80℃ for freezing and storage. The serum isolated above was used to determine the linezolid C_min_. The Concentration of linezolid was determined using a high-performance liquid chromatography-tandem mass spectrometry assay. Quantifying of linezolid was validated over the 0.5–50 µg/mL concentration range with satisfactory accuracies (− 0.59–5.14%), intra-day precisions (≤ 3.45%) and inter-day precisions (≤ 6.99%). The recommended target range of C_min_ for linezolid is 2–7 mg/L^7,8^.

### Primary outcome

The primary outcome of this study was to compare the C_min_ distribution and microbiological efficacy between the two groups. Microbiological efficacy was evaluated only for patients with confirmed Gram-positive pathogenic microorganisms. The evaluation was based on the most recent microorganism clearance result at the end of the treatment course, and the assessment was recorded as documented eradication, presumed eradication, documented persistence, and presumed persistence. Eradication of Gram-positive bacteria is defined as documented eradication and presumed eradication^21^.

### Secondary outcomes

Secondary outcomes included clinical efficacy, 28-day survival, and adverse events. Clinical efficacy was evaluated only in patients with an identified Gram-positive pathogen. The clinical efficacy was evaluated after 7 to 14 days of linezolid administration, and the results were recorded as clinical success or failure^21^. Clinical success was defined as a composite clinical improvement, including: (1) Clinical symptoms: resolution of fever and reduced sputum volume; (2) Inflammatory biomarkers: normalization of white blood cell count (WBC), neutrophil percentage (N%), C-reactive protein (CRP), and procalcitonin (PCT) levels; (3) Respiratory support: successful liberation from mechanical ventilation, transition to conventional oxygen therapy, or cessation of high-flow nasal oxygen; and (4) Radiographic improvement: reduction of inflammatory exudates on chest imaging. 28-day survival and adverse reactions were assessed for all patients. Adverse reactions include LIT, severe LIT and other adverse reactions. According to the causality assessment criteria for adverse reaction, cases which judged to be possible, probable, or certain were recognized as related to linezolid treatment^22^. LIT was assessed during linezolid treatment and within 1 week after discontinuation of the drug. LIT was defined as a platelet count below 112.5 × 10^9^/L (75% lower limit of normal) at any time during therapy in patients with platelet counts above the lower limit of normal (150 × 10^9^/L) at baseline, and severe LIT was defined as a platelet count below 75 × 10^9^/L. For patients with platelet counts below the lower limit of normal range at baseline (75 × 10^9^/L to 149 × 10^9^/L), LIT was defined as a reduction of at least 25% from baseline, and severe LIT was defined as a platelet count below 50 × 10^9^/L^12^. Other adverse reactions refer to other events associated with linezolid treatment^15^, such as headache, diarrhea, nausea, etc.

### Statistical analysis

Continuous variables of normal distribution were expressed as mean ± SD, and comparison between two groups was tested using the independent t-test. Continuous variables of non-normal distribution were described as median with inter-quartile range, and Mann–Whitney test was used for comparison between groups. Categorical variables were expressed as frequencies and proportions (%) and Chi-square test or Fisher’s exact test was used for comparison between groups. Linear correlation analysis or rank correlation analysis was used between the two quantitative variables, and the correlation coefficient was used to assess the correlation between the two variables. Receiver operation characteristic (ROC) curve was plotted to analyze the predictive effect of linezolid C_min_ on gram-positive bacteria eradication. Statistical analysis was performed using SPSS 22.0 (SPSS, Inc., USA) statistical software. Both ROC curve and statistical plotting were performed using GraphPad Prism 9.0 (GraphPad, Inc., USA) software. All p values were two-sided and were considered significant at *P* < 0.05.

## Results

### Patient characteristics

**Fig. 1:**
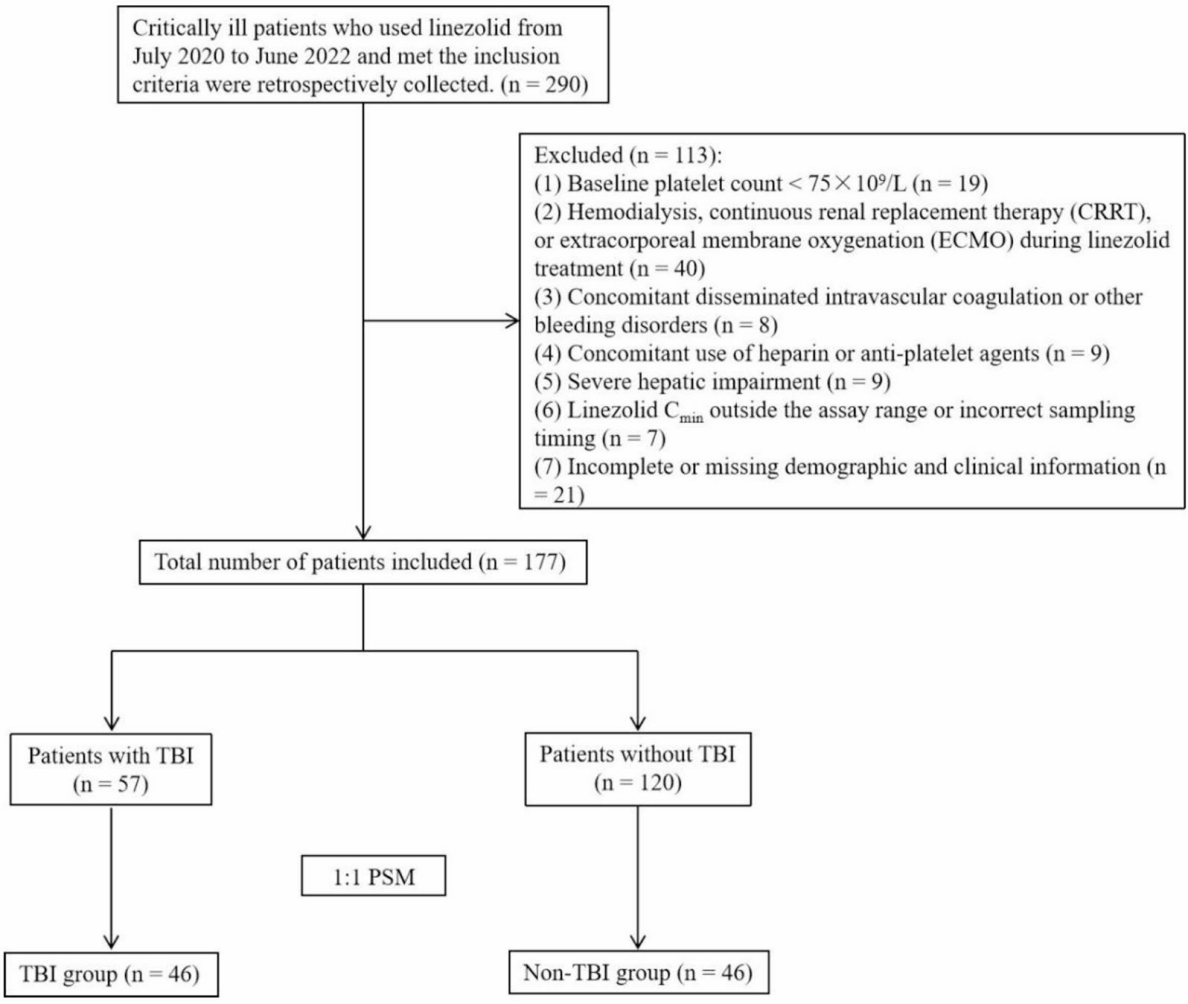
Study flow chart. TBI, traumatic brain injury; PSM, Propensity score matching.

A total of 177 patients who met the criteria were enrolled in the study, including 57 patients with TBI and 120 without TBI. Among all patients, the differences in age, weight, hemoglobin (HB), total bilirubin (TBIL), albumin (ALB) and alanine aminotransferase (ALT) between the two groups in the total study population were statistically significant (*P* < 0.05). Propensity score matching (PSM) was performed between the two groups of patients, after 1:1 PSM, 46 patients with well-balanced baseline levels were enrolled in each of TBI group and non-TBI group (Fig. 1). and there were no significant differences in demographic and clinical characteristics between the two groups (Table 1).

**Table 1:** Demographics and clinical characteristics of the study population before and after PSM. PSM, propensity score matching; TBI, traumatic brain injury; IQR, interquartile range; SD, standard deviation; APACHE II, acute physiology and chronic health evaluation II; *SOFA*, sequential organ failure assessment; CRRT, continuous renal replacement therapy; MODS, multiple organ dysfunction syndrome; WBC, white blood cell; N, neutrophil; hb, hemoglobin; PLT, platelet; CRP, C-reactive protein; PCT, procalcitonin; TBIL, total bilirubin; ALB, Albumin; ALT, Alanine aminotransferase; cr, creatinine; eGFR, estimated glomerular filtration rate.

Characteristics	Before PSM (total population)				After PSM			
	Patients with TBI (*n* = 57)	Patients without TBI (*n* = 120)	Statistics	*P*	TBI group (*n* = 46)	Non-TBI group (*n* = 46)	Statistics	*P*
Demographisc								
Age (year), median (IQR)	61.0 (50.0–70.5)	71.0 (56.0–76.8)	− 2.608	0.009	59.5 (46.0–71.3)	57.0 (48.8–72.0)	− 0.270	0.788
Weight (kg), median (IQR)	65.0 (60.0–75.0)	60.0 (55.0–65.8)	− 3.421	0.001	65.0 (60.0–75.0)	65.0 (59.3–75.0)	− 0.692	0.489
Gender (male), n (%)	46 (80.7)	90 (75.0)	0.706	0.410	38 (82.6)	37 (80.4)	0.072	0.788
Severity of illness								
APACHE Ⅱ score, (mean ± SD)	18.7 ± 10.2	20.0 ± 5.9	− 0.317	0.762	17.1 ± 5.2	19.6 ± 5.0	− 0.873	0.388
SOFA score, (mean ± SD)	6.5 ± 3.7	4.7 ± 2.4	1.550	0.132	6.0 ± 2.6	4.6 ± 2.1	1.293	0.204
CRRT, n (%)	1 (1.8)	8 (6.7)	1.048	0.306	1 (2.2)	2 (4.3)	—	> 0.999
MODs, n (%)	2 (3.5)	12 (10.0)	1.433	0.231	2 (4.3)	6 (13.0)	1.232	0.267
Septic shock, n (%)	3 (5.3)	21 (17.5)	4.937	0.026	3 (6.5)	5(10.9)	0.137	0.711
Respiratory failure, n (%)	26 (45.6)	46 (38.3)	0.849	0.357	21 (45.7)	16 (34.8)	1.130	0.288
Laboratory indexes								
WBC (×10^9^/L), median (IQR)	9.0 (6.9–14.0)	10.3 (7.1–15.6)	− 1.14	0.254	8.9 (6.3–14.0)	8.8 (7.1–12.5)	− 0.06	0.953
N%, median (IQR)	81.2 (71.4–88.1)	84.6 (73.0–91.3)	− 1.078	0.281	82.5 (70.8–88.7)	83.4 (76.4–89.2)	− 0.413	0.680
HB (g/L), median (IQR)	101.0 (89.0–120.5)	90.0 (77.0–107.0)	− 3.13	0.002	99.0 (86.0–108.2)	90.0 (81.5–110.0)	− 1.163	0.245
PLT (×10^9^/L), median (IQR)	207.0 (150–284.5)	193.0 (136.0–299.0)	− 0.52	0.603	200.0 (147.0–289.7)	211.0 (122.0–269.5)	− 0.595	0.552
CRP (mg/L), median (IQR)	56.8 (20.4–111.8)	67.9 (30.1–123.2)	− 1.279	0.201	58.3 (21.4–105.6)	65.3 (22.1–116.8)	− 0.469	0.639
PCT (ng/mL), median (IQR)	0.2 (0.1–0.7)	0.2 (0.1–1.3)	− 0.592	0.554	0.2 (0.1–0.5)	0.2 (0.1–0.6)	− 0.160	0.873
TBIL (µmol/L), median (IQR)	11.7 (7.3–19.0)	8.4 (6.1–12.9)	− 3.071	0.001	11.2 (6.5–16.4)	9.6(6.9–14.7)	− 0.332	0.740
ALB (g/L), (mean ± SD)	34.5 ± 4.9	32.0 ± 4.4	3.376	0.002	33.9 ± 5.1	33.0 ± 3.9	0.900	0.370
ALT (U/L), median (IQR)	39.0 (21.0–59.5)	24.0 (16.0–38.0)	− 2.762	0.006	42.0 (21.7–60.5)	30.5(18.7–45.0)	− 1.570	0.116
Cr (µmol/L), median (IQR)	65.0 (47.8–114.6)	65.5 (45.0–92.7)	− 0.876	0.381	64.1 (46.7–84.5)	57.5 (44.7–71.5)	− 1.054	0.292
eGFR (mL/min/1.73m^2^), median (IQR)	97.5 (53.2–126.0)	94.5 (66.5–110.5)	− 0.524	0.600	102.7 (88.2–128.0)	107.5 (91.3–116.8)	− 0.328	0.743

### Site of infection and pathogenesis

Pulmonary infection was diagnosed in all patients. Nine patients in TBI group had combined intracranial infections, there were no statistically significant differences between the two groups in other sites of infections except for intracranial infections. 29 patients in TBI group and 33 patients in non-TBI group were treated with gram-positive bacteria, and there were no statistical differences in the distribution of bacteria, fungal detection rate, and distribution of the MIC of gram-positive bacteria between the two groups, as shown in Table 2.

**Table 2:** Site of infection and pathogenesis of patients in both groups. TBI, traumatic brain injury; MRSA, methicillin-resistant *Staphylococcus aureus*; MRCNS, methicillin resistant coagulase negative staphylococci; MIC, minimum inhibitory concentration.

Characteristics	TBI group (*n* = 46)	Non-TBI group (*n* = 46)	Statistics	*P*
Sites of infection, *n* (%)				
Pulmonary infection	46 (100.0)	46 (100.0)	–	–
Bloodstream infection	9 (19.6)	7 (15.2)	0.778	0.378
Intra-abdominal infection	2 (4.3)	2 (4.3)	–	> 0.999
Urinary tract infection	4 (8.7)	6 (13.0)	0.015	0.902
Skin and soft tissue infection	3 (6.5)	4 (8.7)	–	> 0.999
Intracranial infection	9 (19.6)	0 (0.0)	9.61	0.002
More than two sites of infection	17 (37.0)	15 (32.6)	1.071	0.301
Cases of Gram-positive bacteria, no. (Strains)	29 (38strains)	33 (41strains)		
MRSA, n (%)	18 (47.4)	14 (34.1)	3.556	0.314
MRCNS, n (%)	10 (26.3)	8 (19.5)		
Enterococcus, n (%)	5 (13.2)	11 (26.8)		
Gram-positive bacillus, n (%)	5 (13.2)	8 (19.5)		
Gram-negative bacteria and fungus, n (%)				
Mixed with Gram-negative bacteria	24 (82.8)	23 (69.7)	1.436	0.231
Mixed with fungus	10 (34.5)	8 (24.2)	0.786	0.375
MIC of Gram-positive bacteria, n (%)				
≤ 1	11 (28.9)	15 (36.6)	0.855	0.661
2	25 (65.8)	23 (56.1)		
4	2 (5.3)	3 (7.3)		

### Clinical outcomes of patients with gram-positive bacterial infections

There was no statistically significant difference in the dosage or duration of linezolid administration between the two groups. The eradication rate of gram-positive bacteria in TBI group was lower than that in non-TBI group, and the difference was statistically significant (69.0% vs. 90.9%, *P* = 0.029). Although the clinical success rate was lower in TBI group compared with non-TBI group, the difference was not statistically significant (72.4% vs. 78.8%, *P* = 0.559). The entire list of clinical efficacy evaluations can be found in Supplementary Table S1 online. LIT and severe LIT occurred in fewer patients in TBI group than in non-TBI group (4.3% vs. 28.3%, *P* = 0.005; 2.2% vs. 17.4%, *P* = 0.035). One patient in TBI group developed diarrhea, while three patients in non-TBI group developed 5-HT_3_ syndrome, intermittent fever, and diarrhea separately. The clinical outcomes of the two groups were shown in Table 3.

**Table 3:** Clinical efficacy and safety evaluation of the two groups. TBI, traumatic brain injury; IQR, interquartile range; LIT, linezolid-induced thrombocytopenia; adrs, adverse drug reactions.

Characteristics	TBI group (*n* = 46)	Non-TBI group (*n* = 46)	Statistics	*P*
Linezolid dosage (mg/kg), median (IQR)	18.5 (15.8–20.0)	17.1 (14.5–20.0)	− 0.835	0.404
Course of linezolid (day), median (IQR)	11.0 (8.0–14.0)	11.0 (8.0–14.0)	− 0.358	0.720
Target therapy, n (%)	29 (63.0)	33 (71.7)	0.791	0.374
Gram-positive bacteria eradication, n (%)	20 (69.0)	30 (90.9)	4.762	0.029
Documented eradication	15 (51.7)	24 (72.7)	–	–
Presumed eradication	5 (17.2)	6 (18.2)		
Documented persistence	6 (20.7)	2 (6.1)		
Presumed persistence	3 (10.3)	1 (3.0)		
Clinical success, n (%)	21 (72.4)	26 (78.8)	0.342	0.559
28-day survival, n (%)	27 (93.1)	29 (87.9)	–	0.676
LIT, n (%)	2 (4.3)	13 (28.3)	7.965	0.005
Severe LIT, n (%)	1 (2.2)	8 (17.4)	4.434	0.035
Other ADRs, n (%)	1 (2.2)	3 (6.5)	–	0.617
Overall adverse events, n (%)	3 (6.5)	16 (34.8)	9.552	0.002

### Analysis of the predictive effect of linezolid C_min_ on the eradication of gram-positive bacteria

**Fig. 2:**
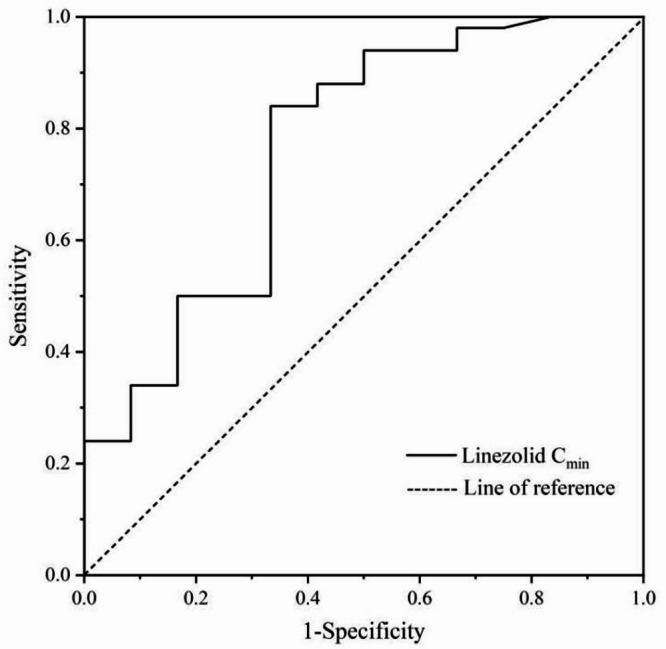
ROC curve for linezolid C_min_ predicting eradication of gram-positive bacteria.

As described above, 29 patients in TBI group and 33 patients in non-TBI group were targeted therapy with Gram-positive bacteria, 20 and 30 patients were eradicated with Gram-positive bacteria at the end of treatment in the two groups respectively. Moreover, these patients were divided into the Gram-positive bacteria eradication group and the non-eradication group and analyzed for the linezolid C_min_ in both groups, the results showed that the linezolid C_min_ was higher in the Gram-positive bacteria eradication group than in the non-eradication group [5.8 (2.4–11.0) mg/L vs. 2.6 (0.6–9.3) mg/L, *P* = 0.024]. The ROC curve of linezolid C_min_ for predicting the eradication of Gram-positive bacteria indicated that: the area under the curve was 0.76 (95% CI 0.63–0.90); the Youden index was 0.51; the calculated cut-off value of linezolid C_min_ was 1.62 mg/L, with the sensitivity 0.84 and the specificity 0.67. The ROC curve was shown in the Fig. 2.

### Effect of traumatic brain injury on linezolid C_min_

**Fig. 3:**
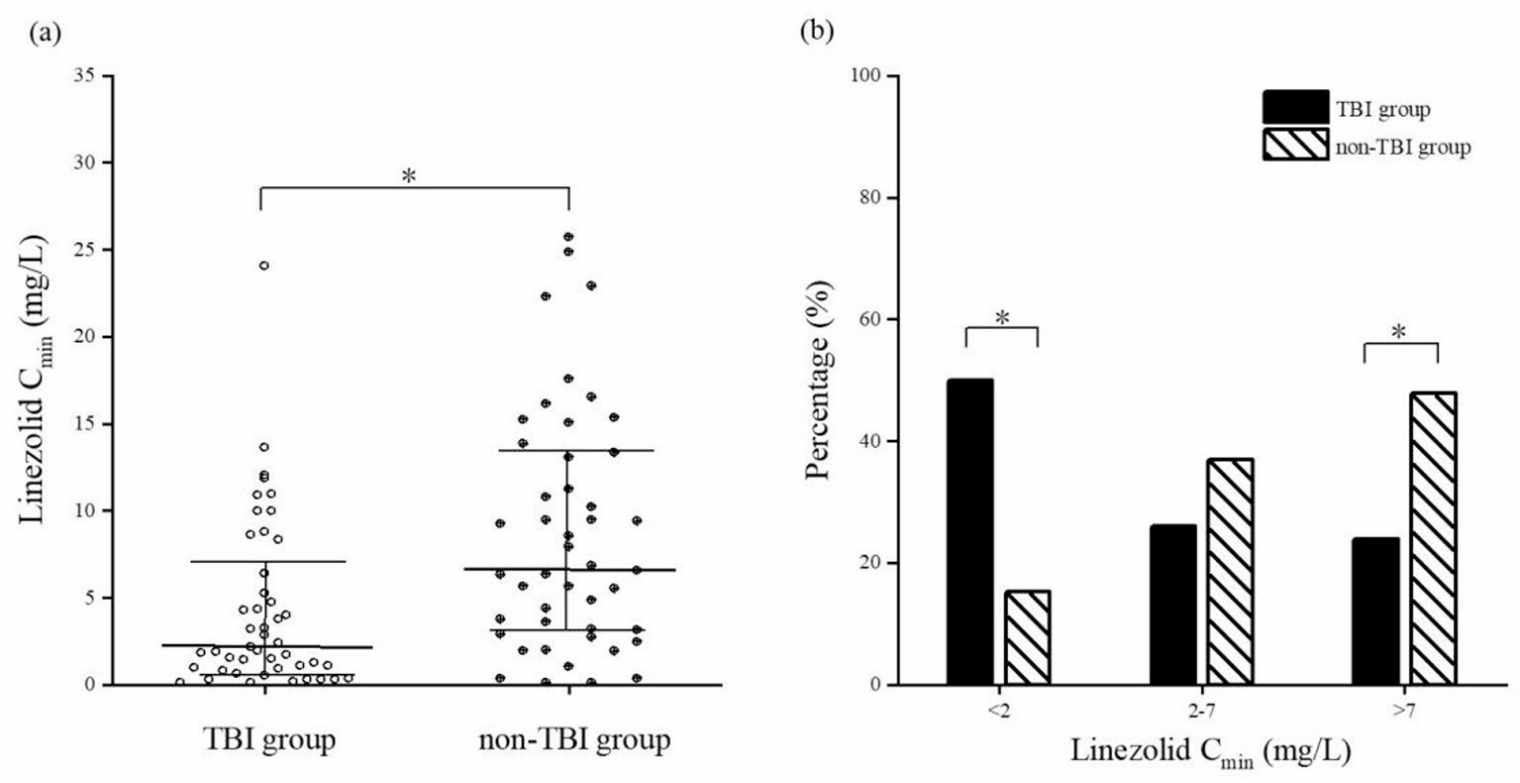
(**a**) Distribution of linezolid C_min_ in the two groups. (**b**) More patients with C_min_ < 2 mg/L in the TBI group and more patients with C_min_ >7 mg/L in the non-TBI group. *Represents a statistically significant difference.

Patients from two groups (46 in each group) were included to analyze the impact of traumatic brain injury on the trough concentration of linezolid. The results showed that patients in TBI group had lower C_min_ than those in non-TBI group [2.06 (0.89, 6.89) mg/L vs. 6.70 (3.09, 13.48) mg/L, *P* < 0.001]. There were more patients with C_min_ < 2 mg/L in TBI group and more patients with C_min_ >7 mg/L in non-TBI group, and the differences were statistically significant (50.0% vs. 15.2%, *P* < 0.05; 23.9% vs. 47.8%, *P* < 0.05). As shown in Fig. 3a,b. There were more patients in TBI group with a trough concentration of < 1.62 mg/L (41.3% vs. 10.9%, *P* = 0.001), similar to the results of patients with linezolid C_min_ < 2 mg/L.

### Analysis of the reasons for lower linezolid C_min_ in TBI group

**Fig. 4:**
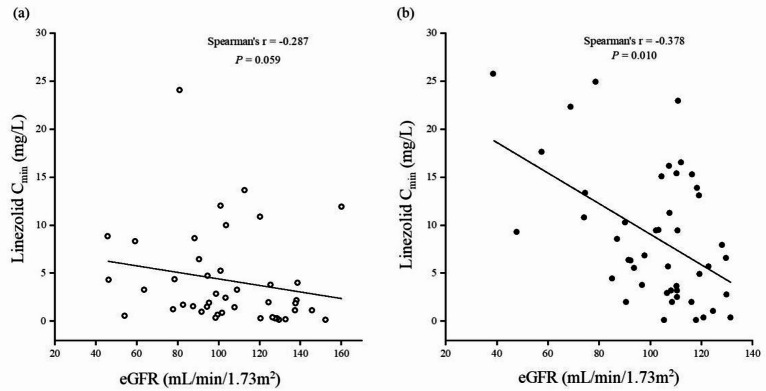
(**a**) Correlation between eGFR and linezolid C_min_ in the TBI group. (**b**) Correlation between eGFR and linezolid C_min_ in the non-TBI group. Statistical significance was assessed by Spearman correlation.

The correlation between linezolid C_min_ and eGFR was analyzed in both groups, and the results showed that C_min_ was negatively corelated with eGFR in non-TBI group (spearman’s *r* = -0.378, *P* = 0.010), however, there was no correlation between C_min_ and eGFR in TBI group (spearman’s *r* = -0.287, *P* = 0.059), shown in Fig. 4a,b.

We statistically analyzed the factors that might affect the C_min_, such as fluid intake and output, diuretic use, etc., and found that more patients were using mannitol and glycerol fructose in TBI group, which resulted in a higher 24 h intake and output before the determination of the concentration, as shown in Table 4.

**Table 4:** Analysis of factors associated with substandard C_min_ in the TBI group. TBI, traumatic brain injury; IQR, interquartile range.

Characteristics	TBI group (*n* = 46)	Non-TBI group (*n* = 46)	Statistics	*P*
Using mannitol and glycerol fructose, n (%)	24 (52.2)	3 (6.5)	18.706	< 0.001
Diabetes insipidus or using desmopressin, n (%)	1 (2.2)	0 (0.0)	0.000	> 0.999
Fluid intake in the 24 h before C_min_ measurement (mL), median (IQR)	3381.0 (2880.0–3926.0)	2755.0 (2382.0–3155.0)	− 2.515	0.012
Fluid output in the 24 h before C_min_ measurement (mL), median (IQR)	2540.0 (1500.0–3540.0)	1550.0 (1250.0–2300.0)	− 2.807	0.005

## Discussion

This two-centre retrospective study found that patients with TBI was at a low exposure, had reduced microbial clearance and clinical treatment success. To our knowledge, this is the first study to demonstrate that hyperosmotic agents indirectly lead to increased urine volume in patients with TBI and is responsible for the difference pathophysiological changes when compared with other populations.

Luque et al.^15^ also reported that the neurosurgical patients had low exposure to linezolid, with plasma C_min_ ranging from < 0.2 mg/L to 2 mg/L, which was lower than TBI group in our study, mainly due to differences in body weight and renal function. The median dose of linezolid in the study by Luque et al.^15^ was 15 mg/kg, which was lower than the 18.5 mg/kg in TBI group in our study. In addition, all patients in the study by Luque et al.^15^ had an eGFR >80 ml/min, with a median eGFR of 158.5 ml/min. In our study, 8 patients in TBI group had eGFR < 80 ml/min, of which 4 patients had eGFR < 60 ml/min, and the median eGFR was 97.5 ml/min, which was significantly lower than in the study by Luque et al.^15^. In another two studies, the plasma C_min_ of linezolid in the neurosurgical patients was 5.35 ± 6.25 mg/L and 5.6 ± 5.0 mg/L, respectively^23,24^, both of which were approximately double that of TBI group in this study. The creatinine clearance rate (97.87 ± 38.55 ml/min and 81.3 ± 39.6 ml/min, respectively) and body weight (58.5 ± 15.6 kg and 67.6 ± 11.8 kg, respectively) in these two studies^23,24^ were not significantly different compared with TBI group in our study, indicating that there were other contributing factors.

 Linezolid exposure was strongly correlated with efficacy and adverse reactions in both groups. In our study, the cut-off value of linezolid plasma C_min_ for eradication of gram-positive bacteria was 1.67 mg/L using ROC analysis. All patients in both groups had pulmonary infections, and most of the gram-positive bacterial samples came from sputum. The concentration of linezolid in pulmonary lining fluid is 8.35 times higher than in plasma^5^, and the effective concentration is more readily achieved in the lung, so the plasma C_min_ cut-off value for microbiological eradication in this study is below 2 mg/L. The proportion of patients with C_min_ >1.67 mg/L in TBI group and non-TBI groups was 63% (29/46) and 89.13% (41/46), respectively, which was closer to the clearance rate of gram-positive bacteria in these two groups. We also observed a significant difference in the incidence of thrombocytopenia between the TBI and non-TBI groups (4.3% vs. 28.3%, *P* = 0.005), which correlated with linezolid exposure. The linezolid C_min_ associated with the development of thrombocytopenia has been reported to be in the range of approximately from 6 to 11 mg/L^7,25^. The incidence of thrombocytopenia was significantly lower in our study compared to those associated with higher linezolid exposure to, as the risk factors of thrombocytopenia are also affected by the duration of linezolid treatment^25^. The occurrence of thrombocytopenia was reported in the linezolid treatment for duration of 10 to 14 days^26^, whereas the median duration of linezolid treatment was 11 days in both study groups, indicating a relatively short duration.

Our study further investigated the factors that influence the plasma concentration of linezolid in TBI patients. Low linezolid exposure has been associated with augmented renal function, overweight or obesity, and younger age in previous studies^10,12,27,28^, however, there were no statistical differences in eGFR, body weight, and age between the two groups in this study. We found a correlation between linezolid plasma concentration and eGFR in non-TBI group, which was not shown in TBI group, suggesting that there are other factors affecting TBI group. Our study found significant differences in hypertonic drug use and urine output between these two groups. Intracranial hypertension and cerebral edema are common complications of TBI^27^, and appropriate hypertonic agents (such as mannitol and hypertonic saline) are often required to reduce brain tissue injury and improve cerebral perfusion^28,29^. In our study, 24 (52.2%) of 46 patients in TBI group were treated with mannitol or glycerol fructose, in comparison only 3 (6.5%) in non-TBI group. Hypertonic agents have a diuretic effect and significantly increase the volume of urine. The median output of urine volume in TBI group 24 h before monitoring was 2540mL, which was significantly higher than that 1550 mL in non-TBI group (*P* = 0.005), suggesting that this may increase the clearance rate of linezolid in TBI group, resulting in low exposure. In addition, in this study, some patients in TBI group had external ventricular drainage (EVD) or lumbar drainage to reduce intracranial pressure and resolve post-traumatic hydrocephalus, and CSF drainage may increase the clearance rate of some drugs, such as vancomycin and meropenem^16,17^. However, the degree of impact on drug clearance varies based on drug permeability. Alexia Chauzy et al.^29^ showed that EVD drainage had a negligible effect on the overall clearance rate of metronidazole, as there is an exchange between CSF and extracellular cerebral fluid, and metronidazole is highly permeable and easily reabsorbed into the cerebrovascular system. Currently, there is a lack of research on the effect of CSF drainage on the clearance rate of linezolid. In the study of Li SC et al.^23^, no effect of CSF drainage on the clearance rate of linezolid was observed, which maybe due to an insignificant difference in the drainage flow of CSF in enrolled patients. Because linezolid and metronidazole have similar hyperpermeability, such as being widely distributed in the central nervous system and only controlled by passive diffusion control^15,20,29,30^, we speculate that external CSF drainage also has little effect on linezolid. In addition, in our study, nine patients in TBI group had central nervous system infection. Due to the destruction of the blood-brain barrier and the blood-cerebrospinal fluid barrier, patients with brain injury and central nervous system infection have increased permeability and may also increase drug distribution as a result^20,30,31^, but drugs with high permeability have been little affected^29^. Yogev et al.^32^ concluded that there was no difference in the permeability of linezolid in children with and without inflammatory meninges.

In this study, the effects of brain injury on plasma linezolid concentration were investigated, and the underlying reasons for this result were also analysed. Limitations of this study include (1) The 24-h clearance of linezolid in urine and CSF drainage have not been studied to explore their effect on linezolid clearance rates. (2) Gram-positive bacteria in this study included MRSA, methicillin resistant coagulase negative staphylococci (MRCNS), enterococci, and positive rods, each with different Clinical and Laboratory Standards Institute (CLSI) sensitivity breakpoints for linezolid (e.g., Staphylococcus spp. MIC ≤ 4, Enterococcus spp. MIC ≤ 2), but further stratified analysis of microbial clearance rates and linezolid plasma concentration breakpoints for different Gram-positive bacteria was not performed. (3) Due to limitations of sample size, the results of this study require further validation.

Consequently, TBI patients are extremely likely to have low exposure to standard doses of linezolid, and patients with comorbid pneumonia have a lower trough concentration but are still at risk of treatment failure. This study did not find an association between low exposure and renal function in TBI patients, but demonstrated a correlation between low exposure and increased urine output due to the use of hyperosmolar agents. It is suggested that this subgroup of patients requires therapeutic drug monitoring of linezolid concentrations and the therapeutic dose can be adjusted by establishing of population pharmacokinetic models.

## Supplementary Information

Below is the link to the electronic supplementary material.

Supplementary Material 1

## Data Availability

The datasets used and/or analysed during the current study available from the corresponding author on reasonable request.

## Abbreviations

ADRs: Adverse drug reactions
ALB: Albumin
ALT: Alanine aminotransferase
APACHE II: Acute physiology and chronic health evaluation II
CLSI: Clinical and Laboratory Standards Institute
C_min_: Trough concentration
CBC: Complete blood count
Cr: Creatinine
CRP: C-reactive protein
CRRT: Continuous renal replacement therapy
CSF: Cerebrospinal fluid
CT: Computed tomography
eGFR: Estimated glomerular filtration rate
EICU: Emergency intensive care unit
EVD: External ventricular drainage
HAP: Hospital-acquired pneumonia
HB: Hemoglobin
ICU: Intensive care unit
IDSA: Infectious Diseases Society of America
IQR: Interquartile range
LIT: Linezolid-induced thrombocytopenia
MIC: Minimum inhibitory concentration
MODS: Multiple organ dysfunction syndrome
MRCNS: Methicillin resistant coagulase negative staphylococci
MRI: Magnetic resonance imaging
MRSA: Methicillin-resistant staphylococcus aureus
N: Neutrophil
PCT: Procalcitonin
PK: Pharmacokinetic
PLT: Platelet
PSM: Propensity score matching
ROC: Receiver operation characteristic
SD: Standard deviation
SOFA: Sequential organ failure assessment
TBI: Traumatic brain injury
TBIL: Total bilirubin
WBC: White blood cell
