# Supplementary material for: Effect of traumatic brain injury on the trough concentration of linezolid in patients with hospital-acquired pneumonia
Source: Sci Rep. 2025 Dec 29;15:44808. doi: 10.1038/s41598-025-28571-9 (PMC12749282; doi:10.1038/s41598-025-28571-9)
Supplement: Supplementary file 1 — Supplementary Material 1 [file 41598_2025_28571_MOESM1_ESM.doc]

| **Characteristics** | **TBI group**  **(Target therapy) (n = 29)** | **Non-TBI group**  **(Target therapy)**  **(n = 33)** | **Statistics** | ***P*** |
| --- | --- | --- | --- | --- |
| Clinical success, n (%) | 21 (72.4) | 26 (78.8) | 0.342 | 0.559 |
| **Normalization of clinical symptoms , n (%)** | | | | |
| Body temperature | 11 (40.7) | 17 (51.5) | 0.693 | 0.405 |
| Sputum volume | 12 (41.4) | 14 (42.4) | 0.007 | 0.934 |
| **Normalization of inflammatory biomarkers , n (%)** | | | | |
| WBC | 4 (14.3) | 11 (33.3) | 2.964 | 0.085 |
| N% | 5 (17.2) | 17 (51.5) | 7.920 | 0.005 |
| CRP | 8 (27.6) | 13 (39.4) | 0.961 | 0.327 |
| PCT | 3 (10.3) | 9 (27.3) | 2.834 | 0.092 |
| **Respiratory support** | | | | |
| *Patients receiving mechanical ventilation, n (%) | 9 (31.0) | 8 (24.2) | 0.385 | 0.550 |
| Successful liberation from mechanical ventilation, n (%) | 3 (33.3) | 4 (50.0) | — | 0.637 |
| Liberation from mechanical ventilation to conventional oxygen therapy, n (%) | 4 (44.4) | 3 (37.5) | — | > 0.999 |
| Failure to liberate from mechanical ventilation, n (%) | 2 (22.2) | 1 (12.5) | — | > 0.999 |
| *Patients receiving high-flow oxygen therapy, n (%) | 17 (58.6) | 20 (60.6) | 0.025 | 0.874 |
| Cessation of high-flow oxygen therapy, n (%) | 13 (17.6) | 16 (80.0) | — | > 0.999 |
| Ongoing high-flow oxygen therapy, n (%) | 3 (17.6) | 4 (20.0) | — | > 0.999 |
| Escalation to mechanical ventilation, n (%) | 1 (6.3) | 0 (0.0) | — | 0.444 |
| **Radiographic improvement, n (%)** | 10 (34.5) | 16 (48.5) | 1.243 | 0.265 |

**Supplementary Table S1**. Assessment of the clinical efficacy in the target treatment population of the two groups. TBI: traumatic brain injury; IQR: interquartile range; LIT: linezolid-induced thrombocytopenia; WBC: white blood cell; N: neutrophil; CRP: C-reactive protein; PCT: procalcitonin. *: The patient was receiving this treatment before taking the medicine.
